# Impact of Cooking on Tuber Color, Texture, and Metabolites in Different Potato Varieties

**DOI:** 10.3390/foods13233786

**Published:** 2024-11-25

**Authors:** Jun Hu, Jinxue Hu, Shaoguang Duan, Fankui Zeng, Shuqing Zhang, Guangcun Li

**Affiliations:** 1 State Key Laboratory of Vegetable Biobreeding, Institute of Vegetables and Flowers, Chinese Academy of Agricultural Sciences, Beijing 100081, China; 2 Shijiazhuang Academy of Agriculture and Forestry Sciences, Shijiazhuang 050041, China; 3 Research Center for Natural Medicine and Chemical Metrology, Lanzhou Institute of Chemical Physics, Chinese Academy of Sciences, Lanzhou 730000, China

**Keywords:** potato tuber, cooking treatment, color difference, texture, metabolome

## Abstract

Potatoes are a globally important crop with high nutritional value. Different potato varieties display notable variations in color, texture, and nutrient composition. However, the influence of cooking on tuber color, texture, and metabolites has not been comprehensively explored. This study evaluated the color and texture of five potato varieties before and after cooking. Cooking significantly altered tuber color, decreased hardness and adhesiveness, and increased springiness, particularly after steaming. The metabolomic analysis of Zhongshu 49 (ZS49) and Shishu 3 (SH3) tubers was conducted using gas chromatography–mass spectrometry (GC-MS) and ultra-high performance liquid chromatography (UHPLC)-MS/MS. GC-MS identified 122 volatile metabolites, with 42 significantly varying between cooking treatments, while UHPLC-MS/MS detected 755 nonvolatile metabolites, 445 of which showed significant differences. Compared to ZS49, SH3 exhibited a marked increase in umami- and flavor-related metabolites, especially after cooking. This study provides new insights into how cooking affects the quality, texture, and metabolite profiles of potato tubers.

## 1. Introduction

Potatoes are a critical global crop, and is rich in carbohydrates, vitamins, minerals, phenolic acids, flavonoids, anthocyanins, and other bioactive substances. In China, fresh table potatoes constitute a significant portion of the potatoes consumed. Typically, potatoes require cooking, which alters their cellular structure and nutritional components, thereby improving their absorption and utilization by the human body [1,2]. For consumers, color and texture are indicators of food quality, with ripeness and sensory properties being key factors influencing preferences, acceptance, and selection [3]. Despite the nutritional importance of potatoes, the effects of cooking on the color, texture, and phytochemical changes in different varieties remain inadequately documented.

Boiling and steaming are common methods for preparing fresh potatoes in China. Unlike traditional home cooking, large food service systems face extended transportation and storage times before cooking. Effective cooking processes are crucial for enhancing the sensory and nutritional properties of potato tubers [4]. Color differences and surface hardening during pretreatment are critical aspects [5]. The color of potato flesh and its variations are linked to the composition and content of pigments, particularly carotenoids and flavonoids [6,7]. Although examining the color differences and texture changes post-cooking is important, few studies have addressed how different cooking methods impact the levels of the relevant chemicals.

High-throughput metabolomics techniques are increasingly utilized in food science for safety [8] and quality assessments [9,10]. Previous studies have focused on the metabolic profiling of potatoes, sweet potatoes, and other root tubers, examining volatile components and gene expression in freshly cut potato shreds and baked potatoes [11,12]. The volatile analysis revealed that alkanes were the most prevalent, followed by alcohols, aldehydes, esters, furans, and quinones. Studies on the Cooperation 88, Shida 6, and Jianchuanhong potato varieties indicated that the alkaloid content increased in damaged samples compared to undamaged ones [13]. However, the impact of different cooking treatments on potato tuber metabolites remains underexplored.

This study examined the effects of boiling and steaming on tuber color, texture, and volatile and nonvolatile metabolites in different potato varieties. The aim of this study was to improve the current understanding of the quality and metabolic alterations in cooked potatoes.

## 2. Materials and Methods

### 2.1. Materials and Cooking Treatment

Five potato varieties—Zhongshu 27 (ZS27), Zhongshu 49 (ZS49), Zhongshuzao 35 (ZS35), Zhongshuzao 39 (ZS39), and Shishu 3 (SH3)—were cultivated using standard agricultural practices at the Chabei Experimental Station (41°29′ N, 115°4′ E), Institute of Vegetables and Flowers, Chinese Academy of Agricultural Sciences, Hebei Province, China, in May 2023. The tubers were harvested in September 2023, stored in the dark until use, and 5 kg of potatoes are prepared for cooking, with an average of 250–300 g per tuber. For traditional cooking, the tubers were sliced into 4–5 mm thick pieces using a slicer. Five potato slices with a uniform thickness and size were selected and boiled in distilled water for 4.5 min. For steaming, the tubers were halved, placed in a steamer, and steamed for 45 min. The cooking times were selected to balance the sensory quality (e.g., texture and color) with nutrient retention. Post-cooking, the color difference and texture properties were assessed. Samples for metabolomics analysis were immediately frozen in liquid nitrogen and stored at −80 °C.

### 2.2. Color Difference Measurements

The color difference was measured using an NR10QC general colorimeter (Sanenchi Technology Co., Ltd., Shenzhen, China), with three parameters L*, a*, and b* being recorded. L* represents brightness, a* stands for red (+) and green (−), and b* stands for yellow (+) and blue (−). The ΔL, Δa, and Δb values represent the difference in the values of L*, a*, and b* between the two samples. The ΔL, Δa, and Δb values were used to calculate the total color difference (ΔE) as follows: $\Delta E=\sqrt{\Delta L^{2}+\Delta a^{2}+\Delta b^{2}}$. Three samples were randomly selected to measure the color difference between raw and cooked potatoes.

### 2.3. Total Starch Content, Amylose Starch Content, and Granule Size Analyses

The total starch content was measured using a total starch assay kit (Boxbio Int. Ltd., Beijing, China). The amylose content was determined using the Megazyme amylose/amylopectin assay kit (Megazyme Int. Ireland Ltd., Wicklow, Ireland). The potato starch granule size was analyzed using the Malvern Mastersizer 2000 particle size analyzer (Malvern Panalytical Ltd., Malvern, UK) [14].

### 2.4. Analysis of Pasting Properties

The pasting properties of the tuber starches were measured using a modified version of the method described by Liu et al. (2023) [14]. Starch samples (2 g) were dissolved in 25 mL of distilled water to create an 8% starch solution. The starch solution was maintained at 50 °C for 2 min, and then heated to 95 °C at a rate of 12 °C/min. It was held at 95 °C for 2.5 min, cooled from 95 °C to 50 °C at a rate of 12 °C/min, and finally maintained at 50 °C for 2 min. This test was conducted in three biological replicates.

### 2.5. Texture Analysis

The potato tuber texture properties were assessed using a CT3 texture analyzer (Brookfield, MA, USA) [14]. Samples with three replicates were compressed to 10.0 mm using a TA3/100 cylinder probe at a rate of 2 mm·s^−1^, with measurements taken at a release force of 1 g.

### 2.6. Metabolite Extraction for UHPLC-MS/MS Analysis

Tissues (100 mg) were ground with liquid nitrogen, and then resuspended in precooled 80% methanol via microwell vortexing. The samples were incubated on ice for 5 min, centrifuged at 15,000× *g* for 20 min at 4 °C, and a portion of the supernatant was diluted with LC-MS-grade water to achieve a final methanol concentration of 53%. The samples were transferred to fresh Eppendorf tubes, and centrifuged again for 20 min at 15,000× *g* and 4 °C. Finally, the supernatant was injected into a UHPLC-MS/MS system for analysis; three biological replicates were tested. 

### 2.7. UHPLC-MS/MS Analysis

The UHPLC-MS/MS analyses were performed using a Vanquish UHPLC system (Thermo Fisher, Germering, Germany) coupled with an Orbitrap Q Exactive^TM^ HF mass spectrometer at Shanghai BIOZERON Co., Ltd. (Shanghai, China). The samples were injected into a Hypersil Gold column (100 × 2.1 mm, 1.9 μm) with a 12 min linear gradient at a 0.2 mL/min flow rate. For positive polarity mode, eluents A (0.1% FA in water) and B (methanol) were employed; for negative polarity mode, eluents A (5 mM ammonium acetate, pH 9.0) and B (methanol) were used. The following parameters were set: 320 °C capillary temperature, 35 psi sheath gas flow, 10 L/min aux gas flow, 60 S-lens RF level, and 350 °C aux gas heater temperature. 

### 2.8. Extraction of Volatile Metabolic Compounds for GC-MS

To each sample, 950 μL of a mixed solution (methanol/chloroform/ddH_2_O, 70:20:5, *v*/*v*/*v*) was added and vortexed for 30 s. Then, 60 μL each of isotopealanine (10 mM) and nonadecanoic acid (0.2 mg/mL) were added as internal standards and vortexed again for 30 s. The tubes were placed in liquid nitrogen for 5 min, and then thawed at room temperature. The samples were ground in a high-throughput tissue grinder at 70 Hz for 2 min, with the process was repeated twice. The samples were centrifuged at 16,000× *g* for 15 min at 4 °C and 800–850 μL of the supernatant was transferred to a new tube. The samples were concentrated to dryness in vacuo. To the samples, 40 μL of a 15 mg/mL methoxyamine–pyridine solution was added, and the samples were vortexed for 30 s and reacted at 37 °C for 90 min. Then, 40 μL of BSTFA reagent was added to the mixture and incubated at 70 °C for 60 min. The samples were centrifuged at 12,000 rpm for 5 min and the supernatant was transferred to the injection vial. For quality control (QC), 20 µL of each sample was used, and the remainder was used for GC-MS detection.

### 2.9. GC-MS Detection

Gas chromatography was conducted using an HP-5 MS capillary column (30 m × 250 μm, 0.25 μm film) at Shanghai Biozeron Biological Technology Co. Ltd. (Shanghai, China), with a helium flow rate of 1 mL/min to separate the derivatives. A 1 μL sample was injected by autosampler in split mode, with a split ratio of 20:1. Injection, interface, and ion source temperatures were set at 280 °C, 150 °C, and 230 °C, respectively. The temperature program involved an initial hold at 60 °C for 2 min, a ramp of 10 °C/min to 300 °C, and a final hold at 300 °C for 5 min. Mass spectrometry was performed using the full-scan method, covering a range of 35–750 (*m*/*z*).

### 2.10. Data Processing and Metabolite Identification

The raw data files were analyzed using Compound Discoverer 3.3 (CD3.3, Thermo Fisher) for peak alignment, selection, and quantification of each metabolite. The metabolites were identified using the mzCloud, HMDB, and LipidMaps databases. Principal component analysis (PCA) was employed to assess overall expression differences between groups and the degree of variation within groups. A univariate analysis using *t*-tests was used to determine statistical significance (*p*-value). Differential metabolites were defined as those with a *p*-value < 0.05 and fold change ≥2 or ≤0.5. Using ggplot2 in the R language, metabolites of interest were filtered using volcano plots based on log2 (fold change) and −log10 (*p*-value).

### 2.11. Statistics Analysis

Data processing was conducted using Microsoft Excel 2019. Principal component analysis (PCA) and Duncan’s multiple range tests (α = 0.05 level), along with Venn diagrams, volcano plots, radar charts, bar charts, and correlation analysis, were performed using R package models (http://www.r-project.org/, 4 April 2024). Heatmap analysis of the compound data was conducted using ImageGP tools (https://www.bic.ac.cn/BIC/#/, 5 May 2024).

## 3. Results

### 3.1. Color Changes in Different Potato Varieties After Cooking 

**Figure 1:**
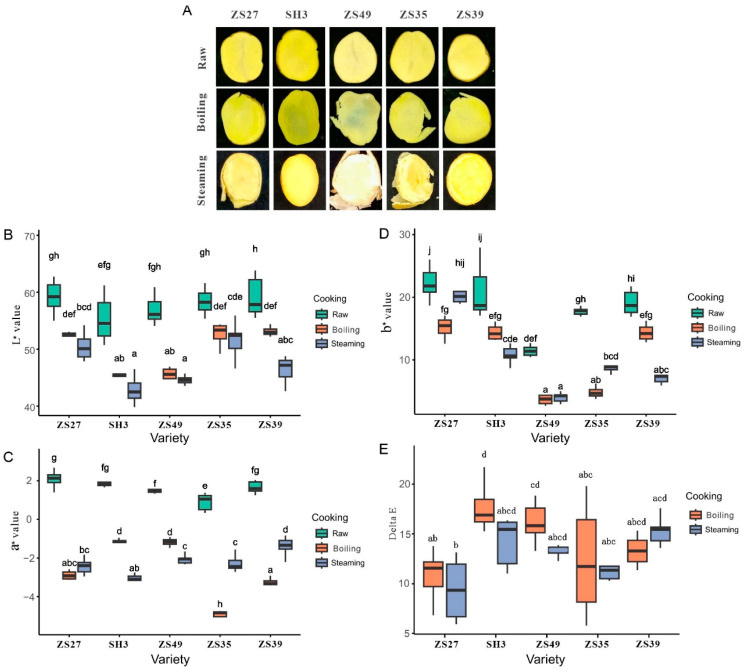
Analysis of color difference of five potato varieties. Morphological observation of different cooking methods (**A**). L* value (**B**), a* value (**C**), b* value (**D**), and delta E value (**E**) of five potato varieties that were cooked using different cooking methods were investigated, *n* = 3. Different letters represent significant differences between different treatment and varieties at *p* < 0.05 level.

The color of the five potato varieties changed significantly after cooking. The L*, a*, and b* values all showed notable reductions after steaming and cooking, compared to their untreated counterparts (Figure 1A–E). Specifically, the L* values (representing brightness) significantly decreased post-cooking, with the reductions in ZS49 and SH3 being more pronounced than in the other three cultivars (Figure 1B). While a* values (red/green) also significantly decreased after cooking in four of the varieties, no significant differences were found among them, except for ZS35 (Figure 1C). The b* values (yellow/blue) exhibited inconsistent changes across the varieties after cooking, with all varieties except ZS27 showing significant decreases (Figure 1D). Finally, no significant differences in total color difference (ΔE) were observed between the boiled and steamed potato varieties (Figure 1E).

### 3.2. Differences in Starch Content, Amylose Content, and Starch Granule Size Among Varieties

1. Significant variations were observed in the starch content, amylose content, and starch granule size across the different potato varieties. The starch granule size distribution was characterized using d10, d50, and d90 values, representing the percentage of granules smaller than specific size thresholds. The starch granule size ranges were 28.37–33.28 μm (d10), 41.82–48.76 μm (d50), and 62.57–70.64 μm (d90). D[4,3] refers to the volume-weighted average particle size, while D[3,2] denotes the surface area-weighted average diameter of the granules. A small difference between D[3,2] and D[4,3] suggests a more regular particle shape and a concentrated size distribution.
2. As shown in Table 1, the potato varieties displayed notable differences in their starch particle size distribution. ZS35 had the largest average particle size (D[4,3]: 50.71 μm; D[3,2]: 46.34 μm) resulting in a smoother texture. In contrast, SH3 exhibited a smaller average particle size (D[4,3]: 47.51 μm; D[3,2]: 43.08 μm) compared to the early-ripening variety ZS35. These findings highlight the impact of starch granule size on the textural properties of different potato varieties.

### 3.3. Starch Viscosity of Different Potato Varieties Showed Significant Differences

The gelatinization temperature is a critical factor in determining the gelatinization properties of starch. Potato starch is characterized by its low gelatinization temperature and rapid viscosity increase, but these properties vary significantly among different varieties. In this study, SH3 exhibited the highest pasting temperature at 73.23 °C (Table 2), which was significantly higher than that of ZS49, ZS39, and ZS35. Moreover, SH3 demonstrated the longest peak viscosity time of 5.91 s. In contrast, ZS49 and ZS39 had the lowest pasting temperatures at 70.75 °C, with ZS49 showing the shortest peak viscosity time of 4.71 s.

Starch viscosity is a key parameter in assessing the gelatinization properties of potato tubers, playing an essential role in evaluating starch quality. A higher starch viscosity typically indicates a softer, waxier texture, whereas a lower viscosity correlates with a softer, sandier texture. Table 2 shows that SH3 had the highest starch viscosity at 2790 BU (Brabender Units), significantly surpassing ZS49, ZS39, and ZS35. In contrast, ZS49 exhibited the lowest starch viscosity at 1956.67 BU. These findings highlight the distinct differences in starch gelatinization and viscosity properties among potato varieties, which can have important implications for their processing and culinary applications.

### 3.4. The Texture of the Tuber Changed Significantly After the Cooking Treatment

**Figure 2:**
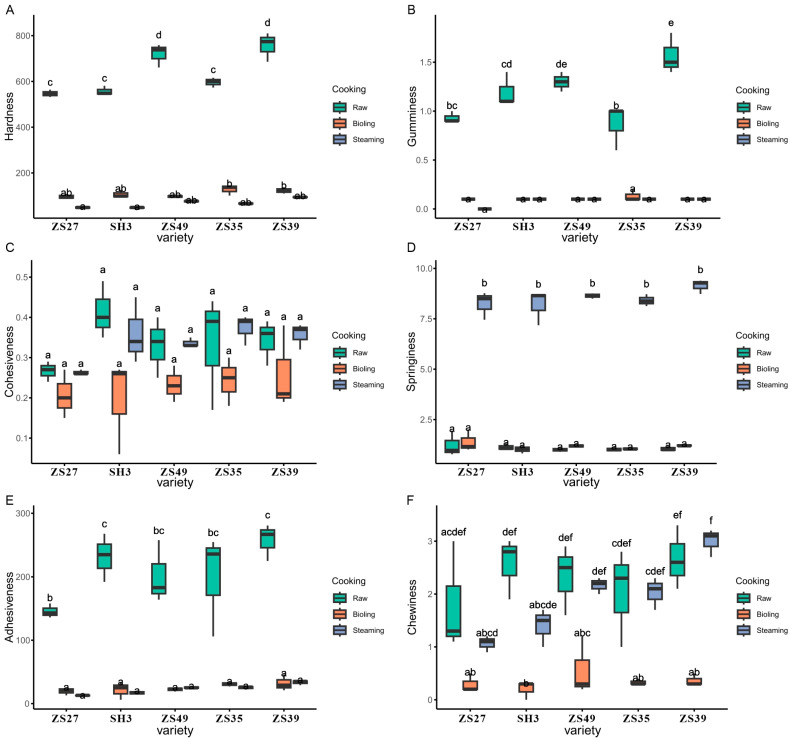
Analysis of tuber texture of five potato varieties. Hardness (**A**), gumminess (**B**), cohesiveness (**C**), springiness (**D**), adhesiveness (**E**), and chewiness (**F**). Different letters represent significant difference between different treatment and varieties at *p* < 0.05 level; *n* = 3.

Cooking significantly impacted the texture of the potato tubers, reducing the hardness, gumminess, and adhesiveness in all five potato varieties (Figure 2A,B,E). These reductions suggest that both boiling and steaming soften the tuber structure, making them easier to chew and less sticky. However, cohesiveness remained unaffected by the cooking treatments, indicating that the internal structure of the tubers remained relatively stable in this aspect (Figure 2C).

Steaming led to a significant increase in springiness (Figure 2D), which implies that the tubers became more elastic and resilient when steamed, enhancing their texture profile. In contrast, boiling resulted in a marked decrease in chewability (Figure 2F), making the tubers less resistant to chewing, likely due to the higher moisture content absorbed during boiling, which softens the tuber structure more than steaming.

These results highlight how different cooking methods can influence specific textural attributes of potato tubers, with steaming improving springiness and boiling leading to softer, more chewable textures. This information is crucial for tailoring cooking methods to achieve the desired texture qualities in various culinary applications.

### 3.5. Correlation Analysis of Starch and Texture-Related Traits

The relationship between starch properties and texture traits was evaluated through a correlation analysis, which revealed significant associations. The amylose content was found to positively correlate with boiling springiness (r = 0.9), peak viscosity (r = 0.51), and raw springiness (r = 0.58), indicating that a higher amylose content contributes to improved springiness in both raw and boiled potatoes as well as to an increase in peak viscosity. However, the amylose content showed a strong negative correlation with peak viscosity time and starch granule size (r = −0.76), suggesting that a higher amylose content is associated with smaller granules and faster viscosity development.

The starch content demonstrated positive correlations with several texture traits, including boiling chewiness (r = 0.81), raw hardness (r = 0.84), steaming hardness (r = 0.7), steaming springiness (r = 0.74), boiling cohesiveness (r = 0.5), steaming adhesiveness (r = 0.52), steaming chewiness (r = 0.57), and raw gumminess (r = 0.69). This suggests that a higher starch content generally enhances texture properties, particularly in terms of hardness, chewiness, and springiness across different cooking methods.

Viscosity parameters, such as viscosity temperature and final viscosity, were positively correlated with setback viscosity (r = 0.49–0.71), peak viscosity time (r = 0.64–0.76), and peak viscosity (r = 0.5–0.74), indicating that starch viscosity parameters are interrelated and contribute to the overall gelatinization behavior of the tubers.

**Figure 3:**
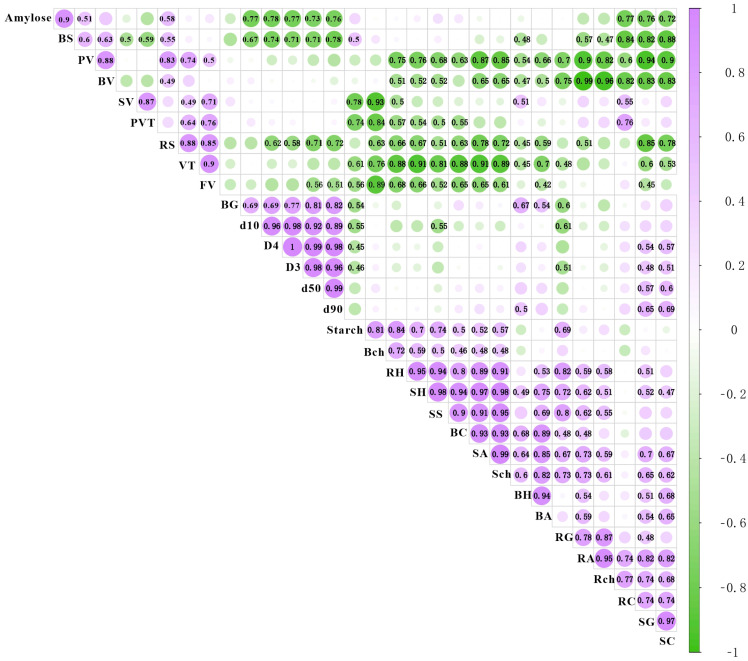
Correlation analysis of relationship between starch content, granule size, starch viscosity, and texture traits. Amylose: amylose content; Starch: starch content; BH: boiling hardness; BG: boiling gumminess; BC: boiling cohesiveness; BS: boiling springiness; BA: boiling adhesiveness; BCH: boiling chewiness; RH: raw hardness; RG: raw gumminess; RC: raw cohesiveness; RS: raw springiness; RA: raw adhesiveness; RCH: raw chewiness; SH: steaming hardness; SG: steaming gumminess; SC: steaming cohesiveness; SS: steaming springiness; SA: steaming adhesiveness; SCH: steaming chewiness; VT: viscosity temperature; PV: peak viscosity; FV: final viscosity; BV: breakdown viscosity; SV: setback viscosity; PVT: peak viscosity time; D4: D[4,3]; D3: D[3,2]. Purple indicates a positive correlation, green indicates a negative correlation, and numerical values are correlation coefficient values that are significant at the *p* < 0.05 level.

Additionally, boiling springiness and raw springiness were closely related to the amylose content, while steaming springiness showed a strong connection to the starch content. Boiling gumminess was positively correlated with starch particle size (r = 0.69–0.82), but negatively correlated with the starch content (r = 0.54), suggesting that larger starch granules contribute to greater gumminess, whereas a higher starch content reduces gumminess (Figure 3).

### 3.6. Analysis of Volatile Metabolites

The GC-MS analysis reveal a total of 122 volatile organic compounds (VOCs) in the SH3 and ZS49 potato tubers before and after boiling and steaming (Table S1), Among these, 42 were identified as differential metabolites (Table S2). These metabolites included 10 benzene and substituted derivatives, 8 aldehydes, 6 alcohols, 4 ketones, 3 esters, 3 phenols, 2 alkanes, and 6 other types of compounds, indicating a broad spectrum of volatile compounds contributing to the potatoes’ flavor profiles.

**Figure 4:**
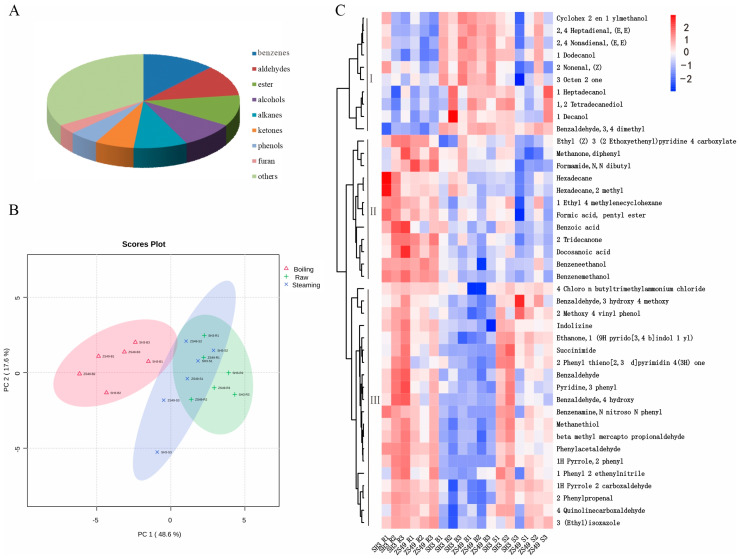
Comparison of volatile compounds in potato tubers of ZS49 and SH3 after using various cooking methods. (**A**) Statistical analysis of volatile compounds. (**B**) PCA score plot illustrating independent experiment replicates. (**C**) Heat map displaying differential metabolites; white blocks show the average relative expression intensity of all volatile compounds, while red blocks show metabolites that were upregulated and blue blocks show metabolites that were downregulated.

The principal component analysis (PCA) revealed distinct differences between the treatments, with the first principal component (PC1) accounting for 48.6% the variance and the second component (PC2) explaining 17.6% of the variance (Figure 4B). The volatile metabolites clustered into three groups based on their concentration changes with the different treatments:

Group I included compounds like 2,4-heptadienal (E,E), 2,4-nonadienal (E,E), 1-decanol, and 1-dodecanol, which were present in low concentrations in raw samples but increased in both boiled and steamed samples. Those compounds may contribute to the development of rich, cooked aromas.

Group II consisted of benzoic acid, formamide, N,N-dibutyl, methanone, diphenyl, hexadecane, hexadecane-2-methyl, benzene ethanol, benzene methanol, and docosanoic acids. These metabolites were more abundant in raw samples but decreased significantly after boiling and steaming, indicating they might be degraded or volatilized during cooking.

Group III featured metabolites such as indolizine, methanethiol, succinimide, benzaldehyde, phenylacetaldehyde, 2-phenylpropenal, and 4-quinolinecarboxaldehyde. These compounds were found at relatively high levels in raw and steamed samples but diminished after boiling (Figure 4C), suggesting that steaming preserves more of these flavor-contributing volatiles.

When comparing SH3 to ZS49, SH3 exhibited a significant increase in specific volatile compounds like 4-ethynylbiphenyl, benzaldehyde-3-hydroxy, benzoic acid, and hexane, indicating that SH3 potatoes had a richer aroma profile (Table S3). This suggests that SH3 potatoes may be more aromatic after cooking, possibly due to the higher concentration of aromatic aldehydes and hydrocarbons. These findings highlight how varietal differences and cooking methods influence the volatile composition and sensory properties of potatoes.

### 3.7. Non-Volatile Metabolite Analysis by LC-MS

**Figure 5:**
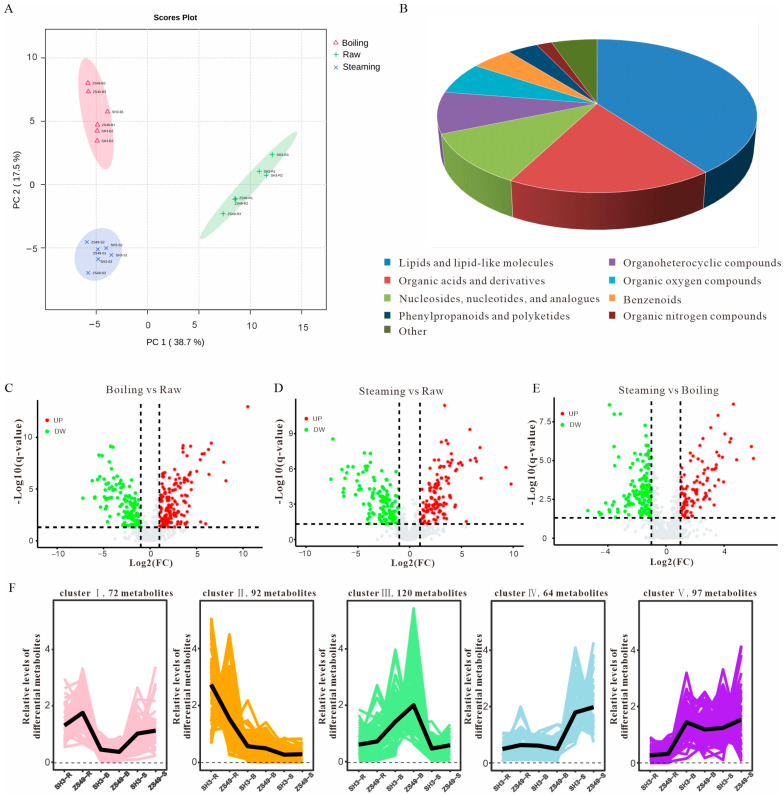
Analysis of nonvolatile metabolites in potato tubers treated with different cooking methods. (**A**) PCA score plot illustrating independent experiment replicates of nonvolatile metabolites. (**B**) Statistics of different metabolite categories. (**C**–**E**) Volcano plot visualization of different metabolites in raw, steamed, and cooked potato tubers. Red spots represent upregulated metabolites and green spots represent downregulated metabolites. (**F**) K-means cluster analysis for differential metabolites.

To investigate the effects of cooking on the nonvolatile metabolites in potato tubers, an untargeted metabolomics analysis was conducted using UHPLC-MS. The principal component analysis (PCA) (Figure 5A) revealed significant differences between the treatments, with ~56.2% of the variance explained by the first two principal components (PC1 and PC2): PC1 accounted for 38.7% of the variance, separating the steamed samples, while PC2 explain 17.5%, distinguished the cooked samples. A total of 755 metabolites were identified (Table S4), and 445 showed differential expression between the treatments (Table S5).

These metabolites were categorized into nine groups based on their chemical properties (Figure 5B): phenylpropanoids and polyketides (16); lipids and lipid-like molecules (177); organic nitrogen compounds (8); organic oxygen compounds (30); organoheterocyclic compounds (39); benzene ring compounds (22); nucleosides, nucleotides, and analogues (49); organic acids and their derivatives (80); and others (24).

The cluster analysis revealed five main patterns of metabolite changes: Cluster I (72 metabolites) had higher concentrations in raw and steamed samples and lower concentrations in cooked samples. This cluster was dominated by nucleotides and organic acids (56.9%). The levels of Cluster II (92 metabolites) were high in raw samples but they decreased significantly after steaming and cooking, with lipids and lipid-like molecules comprising 55.4% of the cluster. The levels of Cluster III (120 metabolites) were low in raw and steamed samples but increased in cooked samples. Lipids, lipid-like molecules, and organic acids represented 45% of this group. The levels of Cluster IV were relatively high in steamed samples but low in raw and cooked samples. This cluster contained nucleosides, nucleotides, and analogues, as well as organic acids and derived metabolites, which accounted for 45% of the cluster. The levels of Cluster V (97 metabolites) were high in steamed samples and low in raw and cooked samples, and included lipids, lipid-like molecules, nucleosides, and nucleotides (45.4%) (Figure 5F). Among these, umami-related compounds such as guanosine monophosphate and uridine monophosphate were low in raw potatoes but they significantly increased after cooking (Table S5), enhancing the flavor profile post-cooking.

Comparing the varieties SH3 and ZS49, 120 nonvolatile compounds were found to differ significantly, with 32 being downregulated and 88 upregulated. SH3 had higher levels of flavor- enhancing compounds such as D-glutamine, DL-glutamine, and gamma-glutamyl glutamine, contributing to its richer taste (Table S6). Conversely, SH3 exhibited lower concentrations of potentially harmful compounds like solanine and free aspartic acid (a precursor to acrylamide), which were more abundant in ZS49 (Table S6). 

## 4. Discussion

Potatoes are a globally cultivated crop and a vital part of the food supply. Starch, the primary component of potato tubers, varies significantly in content and granule size across different genotypes [15]. Variations in physiological and textural properties further distinguish these genotypes [16,17,18]. Starch granules exhibit a wide diameter range (5–85 μm), though their shapes are generally similar [19]. Differential scanning calorimetry showed that the gelatinization transition temperature (69–74 °C) differs among different tuber varieties. The amylose and gelatinization properties of potatoes surpass those of wheat and sweet potatoes, emphasizing their potential in the food industry [20]. The amylose content has been positively correlated with the resistant-starch content and inversely with the glycemic index [21]. Cultivars with higher sensory mealiness scores tend to have lower gelatinization temperatures and a higher amylose content, while potatoes with lower mealiness scores exhibit larger cell sizes and thinner cell walls [22]. 

In this study, the amylose content was negatively correlated with peak viscosity time and starch granule size (Figure 3), which is consistent with previous findings [22,23]. The starch granule size significantly influences tuber texture: smaller granules contribute to a denser, more uniform texture [23]. Among the varieties studied, ZS49 had the highest starch content, while SH3 and ZS35 had a lower starch content. ZS35 also exhibited the largest starch granules, followed by SH3 (Table 1). 

Different cooking methods affect the taste and chemical composition of potatoes in distinct ways. Cooking significantly reduced the hardness, gumminess, and adhesiveness of potato tubers (Figure 2A,B,E). Steaming, in particular, increased springiness (Figure 2D), while boiling significantly reduced chewability (Figure 2F). This indicates that boiling and steaming impact springiness and chewability in opposite ways. Similar effects of cooking have been observed in other root vegetables, such as carrots, where cooking reduces brightness, redness, yellowness, and color saturation [24]. The chemical changes induced by cooking enhance the sensory qualities of potatoes, improving their food safety, taste, and texture [25,26]. For selenium-enriched potatoes, boiling is recommended over frying to retain nutritional quality [27]. Additionally, a study comparing various cooking methods—boiling, steaming, roasting, microwaving, frying, and air frying—on three potato varieties found that steaming and microwaving were the most suitable for preserving the nutritional content in Zhongshu 8 [28]. Despite significant differences in starch content among the five potato varieties, the cooking treatments had a more pronounced effect on texture than the varietal differences. 

Metabolomic analysis was employed to investigate how different cooking methods affect potato metabolites. Higher potato flavor ratings were linked to lower concentrations of specific metabolites [29]. Jiang et al. [30] analyzed how baking and boiling influence purple-fleshed sweet potato metabolites, including starch, soluble sugars, volatile organic compounds, and nonvolatile metabolites. They identified 64 volatile organic compounds, noting that cooking decreased aldehyde levels and increased terpene levels. Among the 871 nonvolatile metabolites, 83.5% remained unchanged post-cooking, with the most significant changes occurring in amino acids, carbohydrates, and phenylpropanoids. In orange-fleshed sweet potatoes (OFSPs), 593 metabolites were identified, with 82.5% remaining unchanged after cooking. Cooking decreased the starch content from 18.15% to 7%, while increasing the soluble sugar content from 11.78% to 39.33% [30]. The carotenoid content decreased by 7–23% depending on the cooking method, with steaming and microwaving being more effective at retaining health-promoting metabolites [30]. Other studies on fresh-cut sweet potatoes showed significant differences in phenylpropanoid biosynthesis, polyphenol oxidase (PPO), and peroxidase (POD) pathways among different varieties [31]. Tian et al. [32] similarly found that steaming and microwaving retained more beneficial metabolites in raw potatoes compared to boiling or frying.

LC-MS analysis revealed that cooking increased the content of health-related compounds like chlorogenic acid and rutin [33]. A study on flavor changes in various steamed potato varieties identified 63 volatile compounds, including 27 aldehydes, 14 alcohols, 12 ketones, 4 esters, 2 furans, and 1 acid, with esters, furans, and acids having the most significant impact on taste [34]. Fresh-cut potato shreds contained the highest levels of alkanes, followed by alcohols, aldehydes, esters, furans, and quinones. Most volatile components, except for hexane, 1,3-hexadiene, and 1,6-octadiene, were reduced several-fold after cooking [11]. While multiple indicators are typically used to assess the effect of different cooking methods, few studies have reported the total changes in metabolite content and composition in potatoes after cooking. Interestingly, no significant differences in volatile metabolites were observed between raw and steamed potato samples, indicating that steaming may help retain volatile compounds better (Figure 4B).

## 5. Conclusions

The results showed that cooking significantly reduced the L*, a*, and b* values in the five potato varieties, leading to marked changes in color. For browning-resistant varieties, this reduction represented a deterioration in color, while for browning-sensitive varieties, it indicated an improvement. The texture analysis indicated that cooking softened the tubers, with boiling notably decreasing chewiness, while steaming enhanced tuber springiness. The starch content, composition, granule size, and viscosity demonstrated varying correlations with texture properties after cooking.

The metabolomic analysis of SH3 and ZS49 tubers identified 445 significantly different nonvolatile metabolites using UHPLC-MS/MS and 42 significant volatile metabolites using GC-MS. SH3 had a notably higher glutamine content in the aroma-related compounds compared to ZS49, with this difference further increasing after cooking. Additionally, SH3 and ZS27 were found to be more suitable for boiling treatments due to their starch and texture properties, while ZS49 and ZS39 were better suited for steaming.

This study provides a comprehensive assessment of how boiling and steaming affect the color, texture, and metabolite profiles of potato tubers, offering valuable insights into the metabolic mechanisms driving changes in potato tuber quality and texture during cooking. These findings contribute to optimizing cooking methods for specific potato varieties, facilitating improved sensory and nutritional qualities in both culinary and industrial applications.

## Figures and Tables

**Table 1:** Starch content, amylose content, and starch granule size of five potato varieties.

Variety	Starch Content (%)	Amylose Content (%)	d_10_/μm	d_50_/μm	d_90_/μm	D[4,3]/μm	D[3,2]/μm
ZS27	15.87 ± 1.15 ^ab^	24.15 ± 4.28 ^b^	29.71 ± 0.16 ^c^	41.82 ± 0.18 ^e^	62.54 ± 2.77 ^c^	44.85 ± 1.40 ^c^	40.8 ± 0.36 ^d^
ZS49	21.71 ± 4.43 ^b^	12.09 ± 1.18 ^a^	31.67 ± 0.12 ^b^	45.95 ± 0.09 ^b^	66.98 ± 0.65 ^ab^	48.11 ± 0.29 ^b^	43.99 ± 0.17 ^b^
ZS39	20.37 ± 1.16 ^b^	20.39 ± 2.73 ^b^	28.37 ± 0.19 ^d^	42.46 ± 0.12 ^d^	64.12 ± 1.52 ^bc^	44.88 ± 0.58 ^c^	40.26 ± 0.22 ^d^
ZS35	11.84 ± 2.02 ^a^	12.36 ± 1.08 ^a^	33.28 ± 0.15 ^a^	48.76 ± 0.06 ^a^	70.64 ± 0.51 ^a^	50.71 ± 0.11 ^a^	46.34 ± 0.18 ^a^
SH3	11.90 ± 1.13 ^a^	9.44 ± 1.18 ^a^	31.59 ± 0.20 ^b^	44.47 ± 0.40 ^c^	66.22 ± 2.16 ^bc^	47.51 ± 1.18 ^b^	43.28 ± 0.57 ^c^

Values are expressed as mean ± standard deviation (SD); *n* = 3. Different letters represent significant differences in the results of different varieties at the *p* < 0.05 level.

**Table 2:** Determination of starch viscosity of five potato varieties.

Variety	Viscosity Temperature/°C	Peak Viscosity/BU	Final Viscosity/BU	Breakdown Viscosity/BU	Setback Viscosity/BU	Peak Viscosity Time/s
ZS27	73.22 ± 0.03 ^a^	5286.33 ± 508.96 ^a^	2657.67 ± 131.86 ^a^	3024.67 ± 406 ^a^	396 ± 29.82 ^ab^	5 ± 0.12 ^bc^
ZS49	70.75 ± 0.05 ^c^	3932.67 ± 135.39 ^b^	1956.67 ± 59.07 ^d^	2296 ± 99.6 ^b^	320 ± 35.79 ^b^	4.71 ± 0.03 ^c^
ZS39	70.75 ± 0 ^c^	3611 ± 78.31 ^b^	2335.67 ± 64.17 ^b^	1697.33 ± 62.92 ^c^	422 ± 45.18 ^a^	5.02 ± 0.1 ^bc^
ZS35	71.27 ± 0.49 ^b^	4114 ± 271.09 ^b^	2181.67 ± 22.23 ^c^	2368 ± 312.5 ^b^	435.67 ± 65.68 ^a^	5.14 ± 0.42 ^b^
SH3	73.23 ± 0.1 ^a^	4145 ± 343.94 ^b^	2790 ± 23.81 ^a^	1833.67 ± 321.9 ^bc^	478.67 ± 38.55 ^a^	5.91 ± 0.14 ^a^

Values are expressed as mean ± standard deviation (SD); *n* = 3. Different letters represent significant differences in the results of different varieties at *p* < 0.05 level.

## Data Availability

The original contributions presented in the study are included in the article/Supplementary Material; further inquiries can be directed to the corresponding author.

## Supplementary Materials

The following supporting information can be downloaded at: https://www.mdpi.com/article/10.3390/foods13233786/s1, Table S1: Identification of volatile metabolites in raw and cooked potato tubers; Table S2: Differentially accumulated volatile metabolites between the raw and cooked potatoes; Table S3: Differentially accumulated volatile metabolites between ZS49 and SH3; Table S4: Identification of nonvolatile metabolites in raw and cooked potatoes; Table S5: Differentially accumulated nonvolatile metabolites between the raw and treated potatoes; Table S6: Differentially accumulated nonvolatile metabolites between ZS49 and SH3.
